# Examining the Impostor-Profile—Is There a General Impostor Characteristic?

**DOI:** 10.3389/fpsyg.2021.720072

**Published:** 2021-09-09

**Authors:** Fabio Ibrahim, Johann-Christoph Münscher, Philipp Yorck Herzberg

**Affiliations:** Department of Personality Psychology and Psychological Assessment, Helmut-Schmidt-University, Hamburg, Germany

**Keywords:** Impostor-Profile, clance impostor phenomenon scale, IPP30, gender, measurement invariance, attributional style, bifactor model, Impostor Phenomenon

## Abstract

The Impostor-Profile (IPP) is a six-dimensional questionnaire measuring the Impostor Phenomenon facets. This study aims to test (a) the appropriateness of a total score, (b) measurement invariance (MI) between gender, (c) the reliability of the IPP, and (d) the convergent validity of the IPP subscales. The sample consisted of *N* = 482 individuals (64% female). To identify whether the scales of the IPP form a total score, we compared four models: (1) six correlating subscales, (2) a general factor model, (3) a second-order model with one second-order factor and six first-order factors, and (4) a bifactorial model with six group factors. The bifactorial model obtained the best fit. This supports the assumption of a total impostor score. The inspection of structural validity between gender subgroups showed configural, metric, and partial scalar MI. Factor mean comparisons supported the assumption that females and males differ in latent means of the Impostor Phenomenon expressions. The omega coefficients showed sufficient reliability (≥0.71), except for the subscale Need for Sympathy. Overall, the findings of the bifactor model fit and construct validity support the assumption that the measurement through total expression is meaningful in addition to the theoretically formulated multidimensionality of the Impostor Phenomenon.

## Introduction

“Shouldn't there be someone better who should be doing this instead of me? It is a question I've quietly asked myself often over the years” (Fridman, 2020). Lex Fridman is a successful scientist in artificial intelligence at the Massachusetts Institute of Technology and has successfully built a YouTube channel and podcast with celebrities such as Elon Musk or Roger Penrose. He responds to a compliment in an interview “I certainly see myself not as successful [...]” (Ask Me Anything with Fridman, 2020). Dr. Fridman himself resonates with the Impostor Phenomenon (IP). His quote illustrates that a lack of internalization of successes characterizes the IP as well as a pronounced self-doubt and a feeling of cheating (Clance and Imes, 1978). The fear of failing in future tasks and subsequently being exposed as an impostor is associated with restricted quality of life and career ambitions (e.g., Neureiter and Traut-Mattausch, 2016; Bernard et al., 2020). Thus, public and scientific interest in the IP has increased substantially over the last 6 years (e.g., Bravata et al., 2019; Mak et al., 2019).

There are currently six instruments available to assess the IP, which have been evaluated in numerous studies and examined for their factor structure (e.g., French et al., 2008; McElwee and Yurak, 2010; Brauer and Wolf, 2016). Mak et al. (2019) questioned the unidimensional scoring method of the existing instruments. The construct's multidimensionality is typically not considered psychometrically, despite having been described with six potential characteristics (Clance, 1985) which vary in composition and occurrence in individuals who feel like “impostors” (Sakulku and Alexander, 2011). The large differences that impostors can exhibit are illustrated by Harvey and Katz's (1985) typology and cannot be fully captured by a single score only. The Clance Impostor Phenomenon Scale (CIPS) also has subscales, however, these were identified through subsequent factor-analytical investigations. Thus, the number of factors and the exclusion of items have not been specified yet (Mak et al., 2019). The possibility of a more differentiated psychometric measurement at the facet level enables a deeper understanding of the IP. Therefore, with the further development of the IPP, we want to provide an instrument that enables differentiated individual diagnostics and global screening concerning impostor tendencies.

In the previous study, we developed the multidimensional Impostor-Profile (IPP; Ibrahim et al., 2020). The construction process of the IPP was inductive by distilling many aspects related to impostorism into several factors. The result is a profile that measures the IP with six subscales named Competence-Doubt, Working Style, Alienation, Other-Self Divergence, Ambition, and Need for Sympathy.

In this study, we firstly want to examine whether the IPP subscales form a general factor and whether a hypothesized IPP general score is related to the most common instrument for measuring the IP. Our inductive approach, with a multidimensional profile followed by a possible total score, allows us to investigate whether the different facets of the IP, represented by the IPP, can be subsumed by a single score. An IPP total score is considered a validation criterion for the existing instruments. Secondly, given the existing disagreement in the current research about gender differences in the IP expression, we examine the measurement invariance between genders and clarify the more fine-grained differences on a subscale level. From our perspective, shifting the question from whether there are general gender differences, to where exactly differences exist at the facet level, may explain the divergent findings. Based on the original formulation of Clance's (1985) theoretical construct, the IP expressions can be of the same level but differ in the composition of the different elements. Finally, we examine the nomological validity of the IPP by considering the relationships with related constructs. Therefore, the IPP is the first initially constructed multidimensional instrument for measuring the IP. The CIPS also has subscales, which, however, were identified through subsequent factor-analytical investigations. Thus, the number of factors and the exclusion of items have not been specified yet (Mak et al., 2019).

### The Impostor Phenomenon

The IP was first described by Clance and Imes (1978) in their psychotherapeutic work. Clance (1985) created the theoretical basis and defined the IP's six core elements, including pre-crastination (extreme over preparation) and procrastination (postponing a task until the last moment), fear of failure, guilt for success, denial of competence and praise, as well as perfectionism. This theoretical basis was later extended by aspects inherent to the IP such as fraudulent ideation, self-criticism, achievement pressure (Kolligian and Sternberg, 1991), fear of being discovered (Leary et al., 2000), as well as discrepancies between self-and reflected appraisals (McElwee and Yurak, 2010). Harvey and Katz (1985) described six different impostor types characterized by a different core element constitution.

The IP is usually referred to as a syndrome in non-scientific articles. However, the term phenomenon is more appropriate because it is not a clinical diagnostic criterion (Bravata et al., 2019). The IP is, in general, a dimensional construct. The individual expression can vary from low to high (Clance, 1985). According to Gravois (2007), 70% of people have experienced impostor feelings at some stage in their lives. These seem to arise, especially during career changes and to decrease with growing professional experience (Prata and Gietzen, 2007). The IP is triggered by contextual factors such as questioning one's expertise, the pressure to publish as a scientist, and comparisons with colleagues (Jaremka et al., 2020).

To date, there is no scientific consensus about gender differences. Clance's original assumption that women are more affected by the IP (Clance and Imes, 1978) has been empirically supported (e.g., King and Cooley, 1995; Cusack et al., 2013) but also contradicted (e.g., Brauer and Wolf, 2016; Rohrmann et al., 2016). The literature shows that younger age relates to higher IP. For example, studies have found robust mean differences when comparing students and working professionals (Cohen's d around 0.50; Brauer and Proyer, 2017, 2019; Neureiter and Traut-Mattausch, 2017). Additionally, the phenomenon occurs across different occupational groups (e.g., Rohrmann et al., 2016; Neureiter and Traut-Mattausch, 2017) and cultures (Sakulku and Alexander, 2011).

### Assessing the Impostor Phenomenon

For the IP assessment, six instruments are currently available. The Harvey Impostor Scale (HIPS; Harvey, 1981) contains 14 items and has a very good to acceptable internal consistency α = 0.64–0.91 (Kolligian and Sternberg, 1991; Holmes et al., 1993). The factor structure of the instrument is still in debate. A four-factorial (Fried-Buchalter, 1992), a two-factorial (Hellman and Caselman, 2004), and a three-factorial structure with the dimensions impostor, unworthiness, and inadequacy (Edwards et al., 1987) have been suggested. Despite the multidimensionality, the HIPS does not calculate subscale values but a total score (Mak et al., 2019).

The most widely used instrument is the Clance Impostor Phenomenon Scale (CIPS; Clance, 1985), showing very good to excellent psychometric properties in various studies α = 0.85–0.96 (Holmes et al., 1993; Simon and Choi, 2018). The CIPS factor structure was also investigated by Jöstl et al. (2012) and Simon and Choi (2018), determining a single factor structure congruent with the CIPS general factor. A two-factorial model was found by French et al. (2008). However, a three-factorial structure (fake, luck, and discount) represents the most widely accepted model (Chrisman et al., 1995; McElwee and Yurak, 2010; Brauer and Wolf, 2016). To establish the three-factorial structure, Kertay, Clance, and Hollande (1991; quoted from Chrisman et al., 1995) excluded items 1, 2, 19, and 20, while Chrisman et al. (1995) excluded CIPS items 1 and 2 to establish a comparable structure. Brauer and Wolf (2016) also determined a three-factor structure with a German version of the CIPS by excluding items 1, 2, 8, and 13.

The Perceived Fraudulence Scale (PFS; Kolligian and Sternberg, 1991) contains 51 items with α = 0.94 for the total score. Factor analysis suggested two factors, Inauthenticity, and Self-deprecation. Like the HIPS and CIPS, no subscales were constructed.

A unidimensional scale is the Leary Impostor Scale (Leary et al., 2000), which includes seven items and assessed the self-perception as an Impostor. The internal consistency is very good to excellent (α = 0.91; McElwee and Yurak, 2007).

The Impostor Self-Concept Questionnaire (Rohrmann et al., 2020) is a recently published 15-item German-language questionnaire. It shows excellent internal consistency (α = 0.93–0.94) and retest-reliability across 4 weeks (*r*_tt_ = 0.77). The Impostor Self-Concept Questionnaire is represented by a total score and assesses the IP by means of a unidimensional trait. The items comprise of: deceiving others about one's abilities, the external attribution of success, the rejection of recognition, and the fear of being exposed as an impostor.

So far, all existing questionnaires assess the IP with a total score. To harness the yet untapped psychometric potential in the theoretical construct's multidimensionality, as manifested in the subscales, the IPP31 was developed. With the IPP, we want to make the inherent elements of the IP measurable, in order to expand psychometric possibilities in science and enable more differentiated diagnostics in a practical context.

### The Impostor-Profile (IPP31)

The construction of the IPP31 (Ibrahim et al., 2020) was based on the theoretical notions by Clance (1985) and Harvey and Katz (1985). The core elements and typical features were extracted and clustered by experts. An initial item pool of 450 items was derived from these clusters, which was reduced to 162 items by several expert rounds. In a pilot study, the pool was further reduced to 62 items by subsequent factor analyses with several samples. Exploratory factor analysis resulted in a questionnaire with 31 items and six factors. This model was tested with a different sample by confirmatory factor analysis and showed a good model fit (GFI = 0.852; AGFI = 0.825; RMSEA = 0.063, 90% CI [0.056–0.065]; CFI = 0.910). However, the Frugality subscale was found to be uncorrelated with any of the other scales (−0.03 ≤ *r* ≤ −0.05). We adapted the instrument by inverting the scale and renaming it to Ambition, as the desire to achieve something extraordinary (e.g., “It is very important to me to create something significant”). This aligns with the theory of the IP, especially the core elements “The need to be special, to be the very best” and “Superwoman/Superman aspects” (Sakulku and Alexander, 2011).

The final version of the IPP31 consists of six scales: Competence Doubt which measures a person's self-doubt, fear of failure, and maladaptive perfectionism. Working Style measures pro- and pre-crastination tendencies by a high or low expression. Alienation describes a feeling of inauthenticity and a tendency toward impression management. Other-Self Divergence measures if the expectations of the environment are perceived as overstraining. Ambition measures the need for success and high self-expectations. The subscale Need for Sympathy measures agreeableness and conflict-averseness. The scales of the IPP31 show sufficient to excellent internal consistency (α = 0.69 and.92), and correlations with the CIPS and the Big Five Inventory indicate the convergent as well as discriminant validity of the instrument.

### Nomological Network of the Impostor Phenomenon

The nomological network of the IP has been explored in various correlation studies, especially by using the CIPS. Following the previous findings, we expect a strong correlation between the IPP total score and the CIPS. This is based on the relation of the largest IPP31 subscale Competence-Doubt with 11 items and the CIPS (*r* = 0.80; Ibrahim et al., 2020), as well as the shared theoretical construct. The attributional style is defined by the dimension's internality (attribution to oneself, up to attribution to circumstances), stability (will be stable over time up to will certainly change), and globality (has just an effect on this scenario up to will affect every other scenario). An external-instable-local attributional style in positive situations characterizes the IP. Brauer and Wolf (2016) found a robust negative correlation between the IP and the attributional style total score in positive situations (*r* = −0.30, *p* < 0.001). The inversed pattern was found between the IP and the attributional style in negative situations (*r* = −0.40, *p* < 0.001).

External attribution of success and the underplaying of one's previous achievements are exhibited as modesty. Leary et al. (2000) showed that study participants high in impostorism expressed lower expectations in their performance only when their responses were public, supporting a strategic nature of impostorism. Therefore, we expect a low correlation between the IPP total score and the Honesty-Humility scale.

In accordance, Schubert and Bowker (2019) showed that impostors are low and fragile in self-esteem. Low self-efficacy (Jöstl et al., 2012; Neureiter and Traut-Mattausch, 2017) and self-handicapping (Want and Kleitman, 2006) are also related to the IP. In addition, the perceived fraudulence, as a central component of the IP (Kolligian and Sternberg, 1991), is assessed by the subscale Alienation. Because of this “facade” that impostors tend to maintain (Clance, 1988), we expected a strong relationship between the subscale Alienation and Self-monitoring, assessed by the Situational Variability subscale.

Fassl et al. (2020) found that social comparison orientation (the frequency of a person engaging in social comparison processes) is positively related to the IP (*r* = 0.45, *p* < 0.001). Accordingly, we expected a large correlation with our subscale Other-Self Divergence.

Furthermore, the IP is associated with depression, negative self-evaluation, psychological distress, and social anxiety, to name but a few (e.g., Chrisman et al., 1995; Vergauwe et al., 2015; Brauer and Wolf, 2016; Rohrmann et al., 2016).

Finally, the IP is relevant in a professional context. Impostors have a lower motivation to lead, lower career ambitions (Neureiter and Traut-Mattausch, 2016), show less organizational citizenship behavior (Vergauwe et al., 2015), and weaker affective commitment (Grubb and McDowell, 2012). Consequently, the IP is a predictor of high perceived workload (Rohrmann et al., 2016) and burnout (Sakulku and Alexander, 2011). Due to these findings, the IP is considered increasingly relevant in coaching (Traut-Mattausch and Zanchetta, 2018) and should be incorporated particularly in coaching programs for young leaders (Kuna, 2019).

### Aim of the Study

The present study aims to investigate the multidimensional structure of the IPP31 further. First, we examined four competing factor models: (1) a six-factor correlated model, (2) a single factor, (3) a second-order factor model, and (4) a single-bifactor model. We hypothesize that a bifactor would explain the covariation of the six dimensions of the IPP31. Secondly, we tested the measurement invariance (MI) to examine gender differences in the IPP31 scores. Finally, we examined the correlations between the hypothesized IPP31 total score with the CIPS to examine convergent validity. We also extended the nomological network of the IPP31 by testing correlations with attributional styles (ASQ), protective self-monitoring, and Honesty-Humility. In the following, we formulate the hypotheses for testing convergent validity and expanding the nomological network according to the correlation guidelines by Gignac and Szodorai (2016; small/medium/large for.10/.20/.30):

**Hypothesis 1a**. The IPP total score is largely related to the CIPS.
**Hypothesis 1b**. The IPP total score is moderately negatively related to the attributional style in positive situations.
**Hypothesis 1c**. The IPP total score is moderately positively related to attribution style in negative situations.
**Hypothesis 1d**. The IPP total score is moderately positively related to the Honesty-Humility scale.
**Hypothesis 1e**. The Alienation scale is largely related to the Situational Variability subscale.
**Hypothesis 1f**. The Other-Self Divergence scale is largely related to the Attention to Social Comparison subscale.

Based on the findings of Ibrahim et al. (2020), we have formulated the following hypotheses for gender differences in the subscales of the IPP.

**Hypothesis 2a**. Females show a higher expression in the subscale Competence Doubt.
**Hypothesis 2b**. Females show a higher expression in the subscale Need for Sympathy.
**Hypothesis 2c**. Males show a higher expression in the subscale Ambition.

## Method

### Participants and Procedure

Data was collected by online survey using Questback (Version EFS Fall 2020). To determine the sample size, we used a sample-to-variable ratio of 1:10 (Osborne and Costello, 2004). Because the bifactor model has more estimated parameters than a corresponding higher-order model (Maydeu-Olivares and Coffman, 2006), we determined a sample of 450 persons as the target size. The original dataset contained *n* = 492 individuals from Germany, of which *n* = 403 individuals were obtained through a commercial panel (Consumerfieldwork GmbH). Further, *n* = 89 individuals were recruited within a German University. Using outlier analysis based on Mahlanobi's distance measure and visual inspection of the data, *n* = 10 individuals were excluded from the data set due to implausible values characterized by a noticeably incoherent response behavior. The final data set contains *n* = 482 persons (64.1% female) with a mean age of 36.96 years ([19, 63], *SD* = 11.41, *Md* = 36.00). Most participants were employed as white-collar workers (*n* = 292), had a general high school diploma (*n* = 172), and did not hold a management position (*n* = 365) (see Online Supplement material for full details on sample characteristics). The dataset for this study can be found in the open science framework: https://osf.io/3yna4/?view_only=c6ebabdbbdca42d4a25367ea655b74f7.

### Instruments

The *Impostor Profile* (IPP31; Ibrahim et al., 2020) comprises 31 items constituting six scales. The instrument uses a visual analog scale with a response range from 1 (“does not apply in any aspect”) to 100 (“applies completely”). The reliability of the six scales ranges from ω_s_ = 0.72 to ω_s_ = 0.92. The IPP is openly accessible in German and in a tentative English version (see Online Supplement for both versions).

The *Clance Impostor Phenomenon Scale* (CIPS; Clance, 1988) is considered the most widely used instrument for measuring impostor expression and contains 20 items. The response scale is a 5-point Likert scale (1 = “not at all true”; 5 = “very true”). In this study, we used the German-language adaptation, which shows a very good internal consistency (α = 0.88; Brauer and Wolf, 2016).

The Revised *Concern for Appropriateness Scale* (CFAS; Lennox and Wolfe, 1984) in German translation, contains 12 items with an internal consistency of α = 0.85. The response scale is five-point 1 (“strongly disagree”) to 5 (“strongly agree”); it includes the two subscales protective variability (α = 0.82) consisting of 6 items and the subscale protective social comparison (α = 0.74) also with 6 items (Laux and Renner, 2002).

The *Honesty-Humility* scale of the *HEXACO-60* (Ashton and Lee, 2008), with 10 items, has an internal consistency of α = 0.72 to.79. The response scale is a five-point Likert scale from 1 (“strongly disagree”) to 5 (“strongly agree”), with a high expression representing honesty, humility, and fairness.

The *Attribution Style Questionnaire* (ASQ; Poppe et al., 2005) contains 16 scenarios concerning the three attribution dimensions internality, stability, and globality. In total, the questionnaire contains eight positive and eight negative situations. The instrument contains two total scores on positive attribution (α = 0.88), with the three attribution dimensions α = 0.74–0.84, and the total score on negative attribution (α = 0.89), with the three attribution dimensions α = 0.73–0.91.

### Statistical Analysis

The statistical model testing, as well as the outlier analysis and the calculation of the correlations, were performed with the software R (R Core Team, 2016) and the packages *psych* (Revelle and Revelle, 2015), *lavaan* (Rosseel et al., 2017), and *semPower* (Moshagen and Erdfelder, 2016). The statistical procedure can be divided into five steps. The post hoc power analysis showed that a misspecified model (with df = 375) corresponding to an RMSEA = 0.05 and α = 0.05 is rejected by a sample size of N = 482 with a power of >99.99%.

First, the data were descriptively inspected for completeness and plausibility. Then, using multivariate outlier analysis by the Mahalanobis distance measure, *n* = 10 individuals were identified with disproportionately larger jumps in the d-squared values. They indicate the distance of an individuum (vector) to the means of all variables in the sample (centroid). Those 10 individuals showed in a detailed inspection an incoherent response behavior and were therefore excluded. Afterward, the sample was examined for descriptive characteristics.

**Figure 1:**
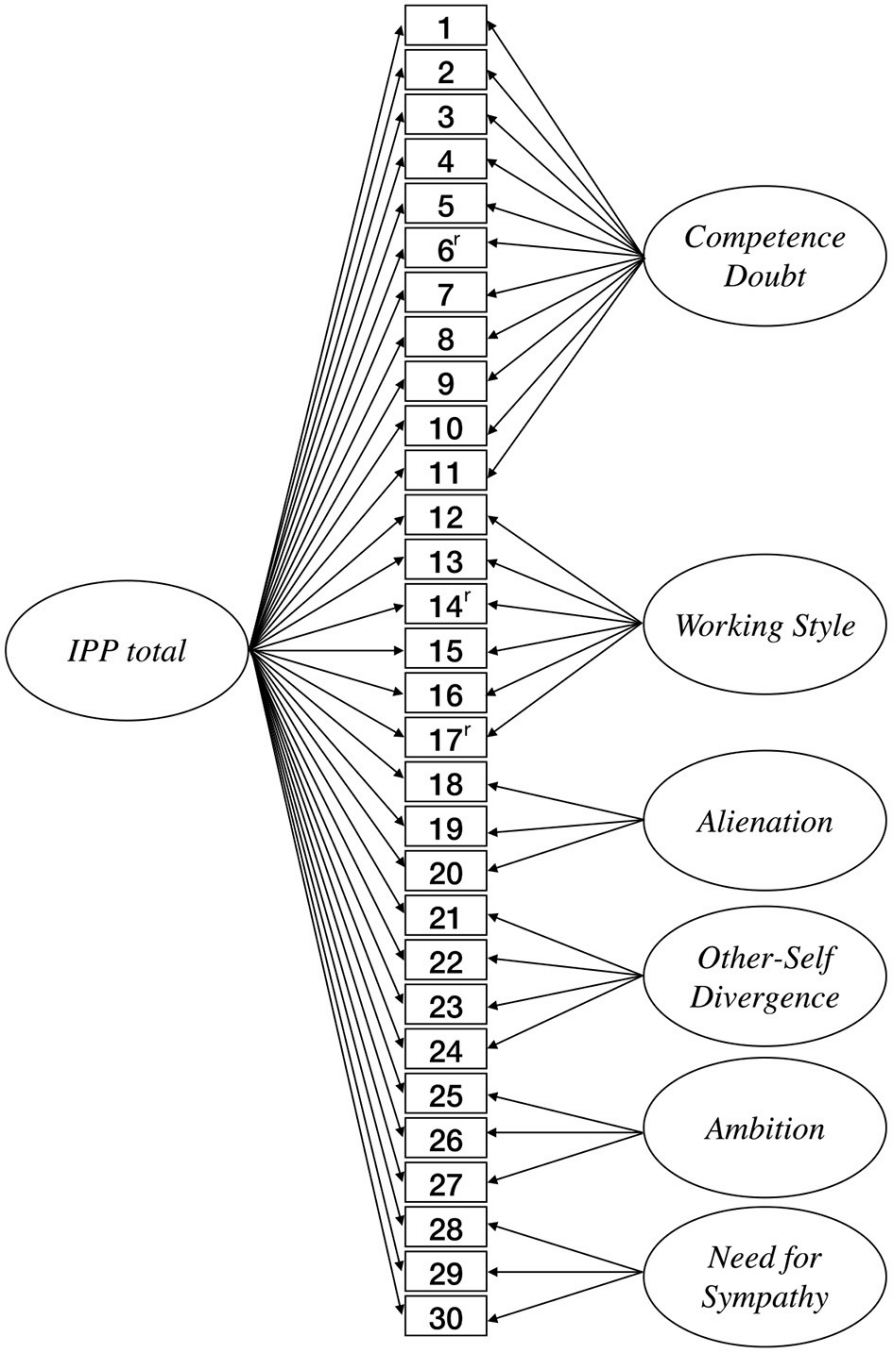
The bifactor model of the IPP30.

In the following, four CFA models were examined: Model (1) as six correlated factor model that represents the subscales of the IPP31 (Supplementary Figure 1), model (2) one general factor that subsumes all items of the IPP31 on a general factor (Supplementary Figure 2), model (3) a hierarchical model with one second-order factor (i.e., total score) and six first-order factors (subscales; Supplementary Figure 3), and (4) a bifactor model with one general factor and six grouping factors (Figure 1). According to Reise et al. (2010), the bifactor model's group factors correlations and the correlation between the bifactor and the group factors were constrained to zero. We chose these four models because they represent different psychometric manifestations regarding measuring the IP. Model (1) represents the IPP as a profile with different subscales and without a total score. Model (2) corresponds to the psychometrical structure of existing IP instruments like the CIPS and the PFS with one total score and no subscales. Mak et al. (2019) questioned this dimensional measurement of the IP because of the multidimensional theoretical formulation of the construct. Model (3) represents a general factor at the second-order level with indirect connections of the items to the higher-order factor. Model (4) represents a general factor for impostorism and group factors that directly explain the variance, which is not explained by the bifactor. Model (4) would be theoretically the most appropriate because the general factor indicates general IP tendencies, and the group factors would explain additional differences. Model (4) would take the original construct into account, indicating impostorism by different compositions of the six core elements. This model would represent the dimensional quality of the general impostor expression using a bifactor. At the same time, the group factors would represent the core elements of the IP (Clance, 1985) and make them differentially measurable.

The robust maximum likelihood estimator (MLR) was used because this method is less dependent on the multivariate normal distribution assumption (Li, 2016). The model evaluation, Hu and Bentler (1999) cut-off values were used: Comparative Fit Index (CFI; ≥ 0.95/0.90 for good/acceptable), Tucker-Lewis Index (TLI; ≥ 0.95/0.90 for good/acceptable), the root mean square error of approximation (RMSEA; ≤ 0.06/0.08 for good/acceptable), the standardized root mean square error residual (SRMR; ≤ 0.10/0.08 for good/acceptable), the Akaike Information Criterion (AIC) and the Bayesian Information Criterion (BIC; lower values indicate better fit).

In the third step, we tested the MI concerning gender for the previously selected model. The CFA was compared in terms of subgroups for males and females to assess the structure's viability across different groups. MI allows to assess whether the latent construct has the same structure across groups (configural measurement invariance; equal number of factors), whether the subgroups attribute the same meaning to the latent construct (metric measurement invariance; i.e., factor loadings are constrained equal across groups), and whether the latent expression of the subgroups is the same based on the response scales (scalar measurement invariance; i.e factor loadings and intercepts are constrained to be equal across groups; Vandenberg and Lance, 2000). According to Chen (2007), we examine measurement invariance by the stepwise model fit comparisons based on CFI (ΔCFI > −0.01) and RMSEA (ΔRMSEA < 0.015).

Next, we computed the reliabilities by means of omega coefficients (Rodriguez et al., 2016). We used four types, namely, one indicating the reliability of the total scale (ω), the reliability of the subscales (ω_S_), the interpretability of the item values as a total score (ω_H_), and the reliability of the subscales after controlling for the variance of the total scales (ω_HS_).

Finally, we examined the construct validity of the IPP31. The suggested correlation guidelines by Gignac and Szodorai (2016; small/medium/large for.10/.20/.30) were used to interpret effect size. The interrelationships of the IPP31 total score and subscales were calculated with the Clance Impostor Phenomenon Scale (CIPS; Clance, 1988), the Concern for Appropriateness Scale (Laux and Renner, 2002), the Honesty-Humility scale of the Hexaco-60 (Ashton and Lee, 2008), and the Attribution Style Questionnaire (Poppe et al., 2005).

## Results

### Descriptive Statistics

Item parameters and reliability coefficients of the IPP31 scales are displayed in Table 1. Item 28 of scale five Ambition (“I would not like to be boss”) was particularly noticeable due to the large standard deviation. A closer look shows that the distribution of item 28 is strongly right-skewed in the subgroup of men (skewness = 0.47; kurtosis = −1.24) and bimodal in the subgroup of women (skewness = 0.12; kurtosis = −1.51). Inspection of scale normal distribution using Shapiro-Wilk-Test shows that all items are non-normally distributed (*p* < 0.001).

**Table 1:** Descriptive statistics, internal consistencies, skewness and kurtosis.

**Scale/Item**	***M* (*SD*)**	**α^a^**	**Skew**	**Kurtosis**	***♂ M* (*SD*)**	***♀ M* (*SD*)**	**Hedges'g**
**Competence doubt**	**39.75 (22.04)**	**0.92**	**0.12**	**-1.09**	**33.95 (21.45)**	**43.02 (21.72)**	**0.42**
1	45.13 (30.9)	0.91	0.03	−1.36	36.84 (29.18)	49.79 (30.85)	0.43
2	45.99 (31.12)	0.91	0.07	−1.38	37.99 (30.47)	50.47 (30.62)	0.41
3	54.39 (30.25)	0.92	−0.16	−1.13	45.90 (30.25)	59.16 (29.17)	0.45
4	32.26 (29.16)	0.91	0.75	−0.64	28.98 (28.72)	34.10 (29.29)	0.18
5	34.41 (29.14)	0.91	0.57	−0.92	29.40 (27.97)	37.22 (29.45)	0.27
6 ^r^	46.10 (29.61)	0.92	0.11	−1.15	42.28 (29.91)	48.24 (29.27)	0.20
7	31.96 (27.62)	0.92	0.66	−0.70	30.91 (26.98)	32.55 (28.0)	0.06
8	36.09 (29.37)	0.91	0.44	−1.07	28.65 (26.49)	40.27 (30.11)	0.40
9	42.26 (30.21)	0.91	0.11	−1.29	36.11 (29.41)	45.72 (30.15)	0.32
10	32.51 (28.54)	0.92	0.66	−0.78	25.32 (24.90)	36.55 (29.67)	0.40
11	36.21 (28.02)	0.91	0.45	−0.88	31.03 (25.97)	39.12 (28.75)	0.29
**Working style**	**45.06 (22.50)**	**0.85**	**0.15**	–**0.83**	**46.55 (23.89)**	**44.23 (21.98)**	−0.10
12	41.03 (29.38)	0.81	0.28	−1.20	40.08 (29.02)	41.57 (29.62)	0.05
13	44.41 (30.20)	0.80	0.20	−1.19	44.96 (30.91)	44.10 (29.84)	−0.03
14 ^r^	55.26 (29.9)	0.87	−0.15	−1.18	58.62 (29.68)	53.38 (29.91)	−0.17
15	39.22 (30.83)	0.80	0.39	−1.17	40.95 (31.64)	38.25 (30.38)	−0.09
16	47.86 (30.14)	0.81	0.01	−1.26	48.42 (30.11)	47.55 (30.20)	−0.03
17 ^r^	42.59 (27.56)	0.85	0.24	−1.05	46.27 (27.98)	40.52 (27.15)	−0.21
**Alienation**	**27.63 (22.30)**	**0.85**	**0.74**	–**0.35**	**28.22 (22.93)**	**27.30 (21.97)**	−0.04
18	23.32 (21.89)	0.85	1.11	0.52	23.87 (22.46)	23.01 (21.60)	−0.04
19	33.28 (28.3)	0.76	0.62	−0.77	33.15 (29.34)	33.35 (27.75)	0.01
20	26.3 (26.09)	0.74	0.97	−0.14	27.64 (26.11)	25.55 (26.09)	−0.08
**Other-Self Divergence**	**31.70 (20.29)**	**0.81**	**0.56**	–**0.19**	**30.40 (20.76)**	**32.43 (20.02)**	0.10
21	32.26 (25.09)	0.75	0.71	−0.34	30.25 (25.05)	33.38 (25.09)	0.13
22	31.73 (25.93)	0.84	0.7	−0.42	32.24 (25.85)	31.45 (26.01)	−0.03
23	28.21 (23.47)	0.71	0.85	−0.11	27.04 (24.0)	28.87 (23.19)	0.09
24	34.6 (27.12)	0.75	0.49	−0.81	32.06 (26.59)	36.03 (27.35)	0.15
**Frugality**	**51.73 (15.56)**	**0.66**	**0.05**	–**0.50**	**47.89 (16.40)**	**56.14 (14.97)**	0.53
25 ^r^	42.95 (27.19)	0.54	−0.25	−0.78	41.86 (27.27)	43.56 (27.17)	0.06
26 ^r^	67.50 (26.72)	0.62	0.65	−0.52	61.73 (28.14)	79.74 (25.37)	0.68
27 ^r^	52.37 (27.74)	0.58	0.19	−0.88	50.75 (28.88)	53.29 (27.08)	0.09
28	44.10 (35.23)	0.64	−0.40	−1.32	37.22 (33.34)	47.97 (35.73)	0.31
**Need for Sympathy**	**68.76 (19.0)**	**0.67**	–**0.57**	**0.20**	**65.34 (19.13)**	**70.69 (18.68)**	0.28
29	68.57 (24.38)	0.36	−0.81	0.14	64.57 (25.19)	70.82 (23.66)	0.26
30	61.68 (26.67)	0.46	−0.52	−0.41	57.96 (27.07)	63.77 (26.26)	0.22
31	76.04 (21.73)	0.82	−1.04	0.65	73.50 (20.95)	77.47 (22.06)	0.18

*The mean value of the items and the standard deviation in brackets are shown for the overall sample, the male (♂) and female (♀) subsample*;

a *Standardized Cronbach's alpha; values for subscales in bold; corrected values when the item is dropped for each item; we used the German version of the IPP31. r, reversed item*.

### Model Comparison of the Impostor-Profile and Fit-Indices

The four competing models are compared based on the fit indices (Table 2). Four additional models M1a – M4a were formulated in which item 28 was excluded due to the bipolar distribution. Model 1 (correlated six-factors) shows a mixed result. Whereas CFI and TLI are below the limits according to Hu and Bentler (1999; CFI = 0.887; TLI = 0.874), RMSEA (0.067), and SRMR (0.080) are in the acceptable range. Model 2 (single-factor) showed inadequate goodness of fit (CFI = 0.615; TLI = 0.588; RMSEA = 0.121; SRMR = 0.102). Model 3 (the second-order factor model with six first-order and one second-order factor) shows mixed results. Also for this model, two indices are below the acceptable limits (CFI = 0.884; TLI = 0.874) and two indices are within the acceptable limits (RMSEA = 0.067; SRMR = 0.082). However, Model 1 has a marginally better goodness of fit (ΔCFI = 0.003; ΔAIC = −13.9). Model 4 (bifactor model with six group factors and one bifactor) exhibits acceptable (CFI = 0.920; TLI = 0.908) to good fit (RMSEA = 0.057; SRMR = 0.052). Furthermore, AIC and BIC are lower than in the other three models. Accordingly, the bifactorial Model 4 has a better goodness of fit than Model 1 (ΔCFI = 0.033; ΔRMSEA = −0.01; ΔTLI = 0.034; ΔSRMR = −0.028) and is used as a basis for further model specification under the exclusion of item 28.

**Table 2:** Goodness-of-fit indices of the models in comparison.

	**Model**	**χ^2^**	**df**	**CFI**	**TLI**	**RMSEA (90%)**	**SRMR**	**AIC**	**BIC**
M1:	Six-factor^31^	1, 172.149	419	0.887	0.874	0.067 (0.062–0.071)	0.08	13,4577.9	134,899.5
M2:	Single-factor^31^	2, 951.490	434	0.615	0.588	0.121 (0.117–0.125)	0.102	136737.1	136,996
M3:	Second-order^31^	1, 200.765	428	0.884	0.874	0.067 (0.062–0.071)	0.082	134,591.8	134,875.8
M4:	Bifactor model^31^	1, 111.732	403	0.920	0.908	0.057 (0.053–0.62)	0.052	134,320.6	134,709.9
M1a	Six-factor^30^	965.651	390	0.910	0.90	0.061 (0.056–0.066)	0.068	129,811.2	130,124.4
M2a	Single-factor^30^	2731.358	405	0.631	0.604	0.121 (0.117–0.125)	0.1	131,965.6	132,216.1
M3a	Second-order^30^	994.614	399	0.907	0.899	0.061 (0.056–0.066)	0.07	129,826.3	130,101.9
M4a	Bifactor model^30^	916.82	375	0.939	0.929	0.051 (0.046–0.056)	0.047	129,597.1	129,972.9

*M1, Six-factor correlated model; M2, Single factor model; M3, Second-order factor model with six first-order and one second-order factor; M4, Bifactor model with six groupfactors and one bifactor; M1a–M4a, Models without item 28; CFI, comperative fit index; TLI, Tucker-Lewis index; RMSEA, root mean square error of approximation; SRMR, standardized root mean square residuals; AIC, Akaike information criterion; BIC, Bayesian information criterion*.

The exclusion of item 28 was considered because of a bimodal distribution in the subgroup of women. The item already attracted attention in the first examination due to its broad distribution (kurtosis = −1.32; Ibrahim et al., 2020). Also, the fit indices' examination shows that the item exclusion contributes to the model fit. Model 4a (bifactor model with six group factors and one bifactor; item 28 excluded; Figure 1) has the overall best fit (CFI = 0.939; TLI = 0.929; RMSEA = 0.051; SRMR = 0.047).^1^ BIC and AIC are also lowest in Model 4a (ΔBIC = −4737; ΔAIC = −4723.5). Accordingly, the bifactorial model with 30 items and six group factors has the best fit. In order to determine the influence of the outlier exclusion on the model fit, we also tested the bifactorial model with the whole data set. When outliers are included, the bifactor model shows no meaningful changes compared to the bifactor model without outliers (ΔCFI = 0.002; ΔTLI = −0.002; ΔRMSEA = −0.001; SRMR = 0.047; ΔAIC = 4463.7; ΔBIC = 4842.5). Theoretically, the bifactorial model also corresponds most closely to the intended use of the IPP. The bifactor model allows the use of an IPP total score, but in addition, the profile and the subscales retain relevance by clarifying additional variance independent of the total score. Thus, a bifactorial model corresponds to the perspective that the same general IP characteristic may differ in the constitution of varying IP inherent elements. Furthermore, the bifactor model enables to examine measurement invariance at the group factor level, where the second-order model only allows to study measurement invariance at the general factor (Reise et al., 2010). In the following, we will use the bifactor model and examine whether there are gender differences in profile expression.

### Measurement Invariance and Analysis of Gender Effects

Due to the divergent findings on the relationship between IP and gender (Cusack et al., 2013; Rohrmann et al., 2016), we used gender to estimate measurement invariance. The configural MI of the bifactor model shows adequate fit indices (Model 1; Table 3). Restricting factor loadings and factor structure across subgroups (Model 2; metric measurement invariance) does not worsen model fit (ΔCFI = −0.005; ΔRMSEA < 0.001). The inspection of the scalar measurement invariance shows that fit worsens according to Chen's (2007) cut-offs: ΔCFI > −0.01, ΔRMSEA < 0.015. Model fit is exceeded based on CFI (ΔCFI > −0.01) while RMSEA remains within the limits (RMSEA = 0.002). Given the lack of scalar invariance, we tested partial scalar invariance by freeing the intercept of item 3 (“Exam situations are very stressful for me. “). Comparing the models for partial scalar measurement invariance and metric measurement invariance indicated sufficient goodness of fit (ΔCFI = −0.004; ΔRMSEA < 0.001; Table 3). Due to the presence of partial scalar invariance, groups can be compared on a latent level (Byrne et al., 1989).

**Table 3:** Standardized factor loadings and Omega coefficients of the bifactor model.

**Item**	**IP**	**CD**	**WS**	**A**	**O-S D**	**Am**	**NfS**
1	0.68	0.63					
2	0.81	0.38					
3	0.47	0.12					
4	0.83	0.10					
5	0.79	0.19					
6 ^r^	0.40	0.32					
7	0.71	−0.02					
8	0.71	0.17					
9	0.70	0.36					
10	0.64	0.22					
11	0.77	0.05					
12	0.56		0.64				
13	0.53		0.74				
14 ^r^	−0.07		0.50				
15	0.41		0.70				
16	0.48		0.60				
17 ^r^	0.20		0.46				
18	0.57			0.42			
19	0.62			0.56			
20	0.64			0.60			
21	0.56				0.52		
22	0.52				0.21		
23	0.66				0.56		
24	0.53				0.55		
25	0.17					0.86	
26	0.11					0.26	
27	0.13					0.74	
29	0.26						0.89
30	0.32						0.68
31	−0.05						0.35
ω (ω_S_)	0.95	0.91	0.82	0.84	0.83	0.71	0.50
ω_H_ (ω_HS_)	0.76	0.20	0.50	0.38	0.33	0.67	0.40

*ω_S_, Omega subscale; ω_H_, Omega hierarchical; ω_HS_, Omega hierarchical subscale; item 28 excluded; Abbreviated IPP30 dimensions; CD, Competence Doubt; WS, Working Style; AA, Alienation; O-S D, Other- Self Divergence; AM, Ambition; and NfS, Need for Sympathy. r, reversed item*.

### Reliability of Subscales and Total Score

Omega coefficients were computed to evaluate the portion of variance attributable to a total score, as well as the reliability of the scales (Table 4). The reliability of the total score is ω = 0.95. The subscales Competence Doubt (ω_s_ = 0.91), Working Style (ω_s_ = 0.82), Alienation (ω_s_ = 0.84), Other-Self Divergence (ω_s_ = 0.83), and Ambition (ω_s_ = 0.71) show acceptable to very good reliabilities. The Need for Sympathy scale (ω_s_ = 0.50) shows comparably low reliability and, accordingly, should not be used as an independently interpretable subscale. The value ω_H_ = 0.77 (Omega Hierarchical) shows that a considerable amount of the composite IPP score is attributable to a general factor (77%). The coefficients omega hierarchical subscale (ω_HS_) indicates a factor's reliability when controlling for the bifactor. Values range from ω_HS_ = 0.20–0.67 and allow the determination of the unique explained subscale variance (i.e., ω_HS_/ω_S_). The explained unique variance by the scales Competence Doubt (22.0%), Working Style (61.0%), Alienation (45.2%), Other-Self Divergence (39.8%), Ambition (94.4%), and Need for Sympathy (80.0%), indicate that the Ambition and Working Style scales, in particular, contribute unique information when controlling for the bifactor.

**Table 4:** Measurement invariance across gender on impostor-profile with 30 items (IPP30).

**Models**		**χ^2^ (*df*)**	**CFI**	**RMSEA**
Model 1:	Configural invariance	1,382.563 (750)	0.921	0.059
Model 2:	Metric invariance	1,476.182 (803)	0.916	0.059
Model 3:	Scalar invariance	1,587.309 (833)	0.905	0.061
Model 3[I_3_]:	Partial scalar invariance	1,522.566 (825)	0.912	0.059
**Model comparisons**		**Δχ^2^** **(Δdf)**	**ΔCFI**	**ΔRMSEA**
M2–M1		93.62 (53)	−0.005	<0.001
M3–M2		111.13 (30)	−0.011	0.002
M3_p_-M2		46.384 (22)	−0.004	<0.001

*The measurement invariance was calculated with the model 4a (item 28 excluded); M1 is a model with a same number of factors and pattern across gender subgroups; M2 with additionally constrain all factor loadings being equal; M3 has first- and second-order factor loadings as well as item intercepts constrained to be equal across gender subgroups; M3[I_3_] as freely estimating intercept of item 3; CFI, comparative fit index; RMSEA, root mean square error of approximation; N_1♂_ = 173, N_2♀_ = 308*.

### Validity Correlations of the IPP30 and Demographic Relations

The previously formulated correlation hypotheses will be used to evaluate the nomological validity of the IPP30. Table 5 reports the correlations between the IPP30 and the ASQ, the Honesty-Humility Scale of the HEXACO-60, the revised Concern for Appropriateness Scale (CFAS), and the Clance Impostor Phenomenon Scale (CIPS). The IPP total score correlates strongly with the CIPS (*r* = 0.78, *p* < *0.0*01; H1a). The IPP total score shows a small negative relationship with the ASQ total score in positive situations (*r* = −0.13, *p* = 0.004). Hypothesis H1b must be rejected. However, further investigation of the ASQ subscale Internality in positive situations shows a negative correlation with the IPP total score (*r* = −0.19, *p* < 0.001). A more detailed investigation shows that the negative relation of the IP and internal attribution in positive situations was stronger for performance (*r* = −0.19, *p* < 0.001) than for social situations (*r* = −0.12, *p* = 0.009). The internal attributional style is moderately correlated with the subscale Competence Doubt (*r* = −0.22, *p* < 0.001) and Other-Self Divergence (*r* = −0.22, *p* < 0.001). The subscales Ambition (*r* = 0.07, *p* = 0.153), Working-Style (*r* = −0.08, *p* = 0.094), and Need for Sympathy (*r* = 0.04, *p* = 0.348) are not related to the internal attributional style in positive situations. Nevertheless, the IPP total score shows a moderate positive correlation with the ASQ total score in negative situations (*r* = 0.41, *p*< *0.0*01; H1c), so hypothesis H1c can be accepted. The effect size for performance (*r* = 0.39, *p* < 0.001) and for social (*r* = 0.36, *p* < 0.001) situations is comparable. An unexpected finding is that the Need for Sympathy subscale is not related to the Honesty-Humility scale of the HEXACO-60 (*r* = −0.02, *p* = 0.678). Hypothesis H1d must therefore be rejected. The Honesty-Humility scale also negatively correlates with the IPP total score (*r* = −0.27, *p* < 0.001) and the CIPS (*r* = −0.21, *p* < 0.001). The Other-Self Divergence scale shows a strong positive correlation with the Attention to Social Comparison subscale of the CFAS (*r* = 0.33, *p* < 0.001), so hypothesis H1f can be accepted. Finally, the Alienation subscale shows a strong positive correlation with the CFAS Situational Variability scale (*r* = 0.62, *p* < 0.001; H1e).

**Table 5:** Correlations of the IPP30 scales and the Attributional Style Questionnaire (ASQ), Clance Impostor Phenomenon Scale (CIPS), HEXACO-60 scale Honesty-Humility and Concern for Appropriateness Scale (CFAS).

		**IPP30**						
	**Subscale (α)**	**IPP Total**	**CD**	**WS**	**A**	**F-S D**	**Am**	**NfS**
	IPP total score (0.92)	1	0.91	0.65	0.70	0.72	0.31	0.35
IPP30	CD (0.92)		1	0.42	0.62	0.64	0.15	0.22
	WS (0.85)			1	0.35	0.31	0.08	0.11
	Alienation (0.85)				1	0.47	0.14	0.11
	F-S-D (0.81)					1	0.13	0.15
	Ambition (0.65)						1	0.24
	NfS (0.67)							1
ASQ	**Positive Situations**							
	Internality (0.78)	−0.19	−0.22	−0.08	−0.17	−0.22	0.07	0.04
	Stability (0.77)	−0.13	−0.19	−0.04	−0.16	−0.14	0.10	0.16
	Globality (0.86)	−0.03	−0.06	−0.02	−0.11	−0.12	0.15	0.16
	Generality (0.88)	−0.08	−0.13	−0.03	−0.15	−0.15	0.15	0.18
	Total (0.90)	−0.13	−0.17	−0.05	−0.17	−0.19	0.13	0.15
	**Negative Situations**							
	Internality (0.80)	0.35	0.34	0.15	0.25	0.34	0.10	0.10
	Stability (0.83)	0.28	0.28	0.10	0.23	0.25	0.02	0.13
	Globality (0.87)	0.40	0.43	0.13	0.30	0.34	0.11	0.12
	Generality (0.90)	0.39	0.41	0.13	0.30	0.34	0.08	0.14
	Total (0.92)	0.41	0.42	0.15	0.31	0.38	0.09	0.14
CIPS	CIPS-Score (0.92)	0.78	0.82	0.35	0.56	0.62	0.17	0.22
	Luck (0.69)	0.52	0.53	0.22	0.39	0.52	0.09	0.09
	Fake (0.67)	0.75	0.80	0.31	0.52	0.58	0.17	0.21
	Discount (0.77)	0.66	0.71	0.26	0.48	0.53	0.05	0.21
	HEXACO-60, Honesty-Humility (0.76)	−0.27	−0.21	−0.16	−0.29	−0.17	−0.24	−0.02
	Concern for Appropriatness Scale (0.88)	0.56	0.50	0.28	0.61	0.37	0.17	0.22
	Situational Variability (0.88)	0.49	0.44	0.26	0.62	0.32	0.13	0.06
	Attention to Social Comparison (0.81)	0.48	0.42	0.23	0.42	0.33	0.18	0.36

*Abbreviated IPP30 dimensions: CD, Competence Doubt; WS, Working Style; A, Alienation; O-S D, Other- Self Divergence; AM, Ambition; and Nfs, Need for Sympathy; Cronbach's α in brackets; |r| > 0.09/0.13/0.15 are related to p < 0.05/0.01/0.001*.

The relationships of the IP with demographic variables show mixed results. The IPP total score correlates slightly positive with gender (*r* = 0.10; Hedges' *g* = −0.21; 95% CI = 0.02–0.39). A more fine-grained inspection of the subscales shows that females show a higher expression in the subscale Competence Doubt (*r* = 0.20; Hedges' *g* = 0.42; 95% CI = 0.23–0.61; H2a) and Need for Sympathy (*r* = 0.14; Hedges' *g* = 0.28; 95% CI = 0.10–0.47; H2b). Males show a higher expression in the subscale Ambition (*r* = −0.10; Hedges' *g* = −0.21; 95% CI = −0.40–−0.03; H2c), while the subscales Working Style (*r* = −0.05; Hedges' *g* = −0.10; 95% CI = −0.29–−0.08), Alienation (*r* = −0.02; Hedges' *g* = −0.04; 95% CI = −0.23–0.15), and Other-Self Divergence (*r* = 0.05; Hedges' *g* = 0.10; 95% CI = −0.09–0.29) are unrelated to gender. The gender differences found in the subscales of the IPP (Ibrahim et al., 2020) could thus be replicated. Age has no significant relationship with the IPP scales and the highest correlation with the subscale Competence Doubt (*r* = 0.06, *p* = 0.203). Interestingly, as assessed by the IPP total score, impostor expression shows a positive correlation with educational attainment (*r* = 0.13, *p* = 0.004), whereas the subscale Ambition has the strongest relation (*r* = 0.14, *p* = 0.003).

## Discussion

In this study, we investigated the multidimensional structure of the Impostor-Profile, the MI between gender, and the instrument's nomological network. Given the inductive construction process from the theoretical derivation of general IP characteristics to the empirical distillation of a profile with subscales, the IPP total score is considered a further step to measure the (multi-)dimensional characteristic. Furthermore, a total IPP score is beneficial for research purposes and applied settings.

To investigate the factor structure of the Impostor-Profile, four CFA models were compared. The bifactor model with six group factors showed the best fit to the data. It allowed examination of the measurement invariance on a group factor level and most appropriately represented the theoretical assumption that the IP consists of different elements but can be measured dimensionally. The four models were also tested in a model extension, excluding item 28. Item 28 was conspicuous within the descriptive analysis of all items, having the largest standard deviation. Closer examination showed that this item is strongly right-skewed for men and bimodally distributed for women. The bifactorial model without item 28 showed the best model performance and was used for further investigation. The lower goodness of fit of the one-factor model (M1) shows that the IPP30 has multiple factors and that the definition of subscales is empirically meaningful. This empirical finding is supported by the six core elements of the IP theoretically formulated by Clance (1985).

The subscales Ambition and Need for Sympathy are uncorrelated with the CIPS. Nevertheless, both subscales represent parts of the theoretical construct. Ambition, the desire to achieve something extraordinary, is evident in the IP's characteristic, “the need to be special/ the very best.” The subscale Need for Sympathy, the requirement to be liked by others, is anchored in the core element “fear and guilt about success,” which implies that impostors are afraid of rejection by others (Sakulku and Alexander, 2011). Both scales represent the less socially valued subconstructs that are inherent to the IP but uncaptured by the CIPS, adding a possible psychometric advantage to the IPP30.

To the best of our knowledge, this was the first study to address gender differences in the IP by testing MI across males and females. The examination of MI shows that configural, metric, and partial scalar measurement invariance can be assumed across genders. For the research context, no distinction needs to be made between genders, as the latent construct and the attribution of meaning to the latent construct are the same across genders. Nevertheless, scalar measurement invariance cannot be assumed because limits were exceeded in the model comparison (Chen, 2007). Consequently, mean differences between gender must be taken into account, and norms must be provided for both genders, especially for diagnostic purposes.

The examination of the omega-coefficients supports the subscales and the bifactor reliability. Contrary to Cronbach's α, the omega coefficient does not assume essential tau-equivalence and is considered a more general estimator of reliability (Hayes and Coutts, 2020). Even though the Need for Sympathy scale's reliability is significantly lower (ω_s_ = 0.50; α = 0.67), this short-scale expression should still be considered for practical applications since the Impostor Profile application lies apart from clinical or aptitude diagnostics (Ziegler et al., 2014). In practical use, however, minor measurement differences in the Need for Sympathy scale should not be over-interpreted, for example, in a follow-up with several measurement time points. Accordingly, it should be interpreted as a subscale with caution and could be used exploratorily. Despite this, the variances elucidated by the subscales when controlling for the bifactor are sufficient (22.0–94.4%) for the subscales to be considered meaningful. Thus, the IPP's merit is that it allows examining both the Impostor-Profile and a total score.

This study showed that women had just slightly higher IP expressions by total scores. This result supports similar findings in previous research (Cusack et al., 2013). Examining the IPP subscale differences allows for a further understanding of the gender differences at the construct's facet level. Women showed higher scores in the subscales Competence Doubt and Need for Sympathy. In particular, the feeling of being incapable, measured by the Competence Doubt subscale, is a central element of the IP. The higher expression within the Need for Sympathy scale could also result from higher self-doubt, indicated by the subscale Competence Doubt, leading to an increased need for social support. These characteristics may result in strategies to compensate for self-perceived performance deficits through social relationships. Men showed higher scores in the subscale Ambition. Possibly men are more inclined to compensate for impostor tendencies with evidence of their competence, such as socially recognized successes or titles. The subscales Working Style, as the tendency to pro- or precrastination, Alienation, as the feeling of phoniness, and Other-Self Divergence, as the perception of excessive expectations by others, show no association with gender. Overall, the study on MI shows that gender is a minor predictor of the general IP expression. Nevertheless, this study emphasizes that the IP is a construct with several facets. Therefore, considering the subscale level can provide a deeper understanding regarding gender differences and different interventional nuances for both genders.

Correlation analyses largely supported the convergent validity of the total score and the subscales. This study extended the ‘big five based'-nomological network of the IPP subscales, which we examined in the previous article (Ibrahim et al., 2020). The strong correlation of the IPP total score with the CIPS score shows that both scales measure a substantially similar characteristic despite the different construction processes and indicate the convergent validity of the IPP total score. As expected, we found a positive association between the IPP30 and the internal-stable-global attributional style in negative situations. The correlations of the IPP total score and the attribution style in negative situations align with previous findings on the correlation of the CIPS and the attribution style in negative situations when depression is not controlled (Brauer and Wolf, 2016). However, the relationship between the IPP total score and attributional style in positive situations was lower than hypothesized.

Nevertheless, the investigation of the internal attributional style in positive situations shows that impostors attribute success less internally and stably. However, the effects are smaller than expected compared to the findings of Brauer and Wolf (2016), but higher than those of Cozzarelli and Major (1990), who found that impostors do not differ in affective response to subjective success. The subscales of the Impostor-Profile show the expected relationships to convergent instruments. The Other-Self Divergence subscale, about the perception of expectations from the external environment, showed a strong positive relationship with the Attention to Social Comparison subscale. The Alienation subscale, which asks about the feeling of inauthenticity, is strongly related to the Situational Variability subscale. Both scales indicate pretense in social situations. The Need for Sympathy subscale did not show the expected correlation with the Honesty-Humility scale. Nonetheless, this can also make sense since these characteristics are derived from different domains. Need for Sympathy (“It is important to me to be liked”) asks about the desire to be liked, whereas Honesty-Humility (inverted: “If I want something from someone, I will laugh at that person's worst jokes.”) differs significantly in the aspect of honesty. However, behavior that is demonstrated in order to be liked may be dishonest. Furthermore, the negative correlation between Honesty-Humility and IPP could be due to the tendency of Impostors to downplay their abilities and character strengths (Clance, 1985). A method to investigate this possible bias could be the method of peer rating, in which the correlation of assessments by associates is determined. This method could allow us to investigate biases in self-assessment, especially of positive characteristics of impostors. The negative correlation of the Alienation Scale, which measures a person's feeling of inauthenticity and fraudulence, with Honesty-Humility, supports the subscale's convergent validity.

Considering the findings regarding the bifactor model's reliability and validity, the use of an IPP total score for assessing a general impostor characteristic is supported. The IPP30 total score and subscales showed acceptable to very good reliability, although Need for Sympathy should be used with intent to assess a content valid characteristic. Further, we provided evidence to the nomological network of the IPP30. Overall, the IPP30 is a multidimensional instrument for the measurement of the IP, which at the same time allows the determination of a general impostor expression.

Overall, the IPP30 is a multidimensional instrument for the measurement of the IP, which also provides a general impostor expression. The Impostor-Profile subscale constellation reveals the complexity and self-sustaining quality of the IP. The application of the IPP30 makes it possible to assess aspects of internal psychological beliefs and ideas in more detail, so that these can be addressed through specific interventions. This diagnostic depth constitutes the increment of the Impostor-Profile.

### Limitations

*One* limiting factor of this study is the imbalanced gender distribution because women represent the larger subgroup. This imbalance may have biased the MI results and should be tested with a larger and more balanced subgroup distribution. Most individuals in the sample were salaried workers. This occupational group-specific overrepresentation may have reduced the generalizability of the results. Future research should also investigate whether different occupational groups differ in terms of their IP expression. In addition, the sample was exclusively German. Therefore, an English version of the IPP should be validated to increase the number of users. The survey was conducted as an online survey, so the study conditions could not be controlled, and *n* = 403 individuals were commercially compensated participants who were externally motivated. Furthermore, the research data was exclusively self-reported data, which only allows for a partial picture, especially regarding the IP and the implications for distorted self-perception. Despite the use of control scales and immediate survey exclusion for incorrect responses, individuals may have responded in an unconscientious manner. In the future, the construct validity of the IPP should be further specified, especially by testing the discriminant validity, which has not been considered so far. In addition, the bifactorial structure should be tested in an independent sample to check replicability.

### Perspectives

This study supports the assumption that the Impostor-Profile has a general factor. The high correlation of the CIPS and the IPP total score serves as an argument for a general impostor characteristic despite the theoretical construct's multidimensionality.

Future research should use the IPP to examine subscale expressions in addition to a general IP expression. A combined examination of the IPP total score and the subscales provide the most significant insights. However, the IP total expressions could be associated with different subscale constellations. The IP as a broad construct can thus be explored more specifically in its facets and its interaction. Besides, the profile with the subscales can be used to empirically test the typology of the impostor proposed by Harvey and Katz (1985). Another research question would be whether there is a detectable distinction between true impostors and strategic impostors by different subscale expressions within the IPP30. True impostors perceive that others overrate them, and they have a fear of being exposed. Strategic impostors communicate self-doubts as a self-presentational tactic to lower others' expectations and to appear modest (Leary et al., 2000).

Another fascinating point would be the further development of the IPP30 as an informant assessment form that would allow comparing the perceived inauthenticity and the expectation of the environment with the impostor's perceived environmental expectation. The comparison of self-and informant reports, especially in the Alienation, Other-Self Divergence, and Competence Doubt subscales, would provide further insight into the IP.

Overall, the high correlation of the IPP30 and CIPS supports both instruments' validity and the IP's unidimensional measurability. Furthermore, the IPP30's extension of a total score enables a general assessment of the impostor characteristic. The use of the IPP30, including subscales and total score, allows a more detailed investigation of the phenomenon in science and practice and could stimulate further research questions and intervention methods.

## Data Availability Statement

The datasets presented in this study can be found in online repositories. The names of the repository/repositories and accession number(s) can be found below: https://osf.io/3yna4/?view_only=c6ebabdbbdca42d4a25367ea655b74f7.

## Ethics Statement

Ethical review and approval was not required for the study on human participants in accordance with the local legislation and institutional requirements. The patients/participants provided their written informed consent to participate in this study.

## Author Contributions

This article was written in joint collaboration. FI had the initial study idea, with PH and J-CM assisting with the final design. FI performed the data collection. FI performed the data analysis in collaboration with J-CM. FI made the data interpretation, with PH providing essential suggestions for improvement. FI prepared the first draft article and improved it through three feedback cycles with improvements from J-CM and PH. Critical revision has been done by PH. The final submission has been done by FI. All authors contributed to the article and approved the submitted version.

## Conflict of Interest

The authors declare that the research was conducted in the absence of any commercial or financial relationships that could be construed as a potential conflict of interest.

## Publisher's Note

All claims expressed in this article are solely those of the authors and do not necessarily represent those of their affiliated organizations, or those of the publisher, the editors and the reviewers. Any product that may be evaluated in this article, or claim that may be made by its manufacturer, is not guaranteed or endorsed by the publisher.

## Footnotes

^1^We further examined the model with regard to Modification Indices. We found that surface features mention the same word (e.g., “task”) in items 13 and 15. In four cases, items have the same wording and are in the same subscale. When their error variances are allowed to correlate, model fit improves compared to Model 4a (ΔCFI =.007; ΔTLI =.008; ΔRMSEA =.003).
