# Supplementary material for: Examining the Impostor-Profile—Is There a General Impostor Characteristic?
Source: Front Psychol. 2021 Sep 9;12:720072. doi: 10.3389/fpsyg.2021.720072 (PMC8458651; doi:10.3389/fpsyg.2021.720072)
Supplement: Supplementary file 1 [file Data_Sheet_1.pdf]

Supplementary Material

Table S1

Sample Description by Age, Occupation, and Educational Level.

		Male (%)	Female (%)	Total (%)
		173 (36)	309 (64)	482 (100)
Age	18 - 23	24 (5)	50 (10)	74 (15)
	24 - 29	27 (6)	51 (11)	78 (17)
	30 - 35	31 (6)	42 (9)	73 (15)
	36 - 41	31 (6)	66 (14)	97 (20)
	42 - 47	18 (4)	39 (8)	57 (12)
	48 - 53	20 (4)	22 (5)	42 (9)
	54 - 59	20 (4)	37 (8)	57 (12)
	60 - 70	2 (<1)	2 (<1)	4 (<1)
Occupation	Pupil	0 (0)	0 (0)	0 (0)
	Student	24 (5)	46 (10)	70 (15)
	Pensioner	0 (0)	1 (<1)	1 (<1)
	Unemployed	0 (0)	0 (0)	0 (0)
	Skilled worker	21 (4)	8 (2)	29 (6)
	Salaried worker	96 (20)	196 (41)	292 (61)
	Self employed worker	11 (2)	20 (4)	31 (6)
	Freelancer	6 (1)	12 (2)	18 (3)
	Civil servant	8 (2)	6 (1)	14 (3)
	Academic	0 (0)	7 (1)	7 (1)
	Soldier	8 (2)	12 (2)	20 (4)
Education level	No educational attainment	0 (0)	0 (0)	0 (0)
	Certificate of secondary Education	22 (5)	17 (4)	39 (9)
	Intermediate school leaving certificate	24 (5)	60 (12)	84 (17)

General higher education qualification	58 (12)	114 (24)	172 (36)
Bachelor's degree, Master's degree	60 (12)	111 (23)	171 (35)
Doctorate or higher	9 (2)	7 (1)	16 (3)

Note: The percentages have been rounded to whole numbers; the german sample was recruited in the period from October to November 2020.

Table S2

Items of the IPP30 in the original German version and an tentative English translation.

	Deutsch	English
	**Kompetenz-Zweifel**	**Competence Doubt**
1	Trotz vergangener Erfolge habe ich starke Versagensängste.	Despite former successes, I have a strong fear of failure.
2	Ich denke häufig, dass meine Fähigkeiten nicht ausreichen.	I often think that my skills are not enough.
3	Prüfungssituationen sind für mich sehr belastend.	Exam situations are very stressful for me.
4	Aus Angst zu versagen, verliere ich öfter den Spaß an der Arbeit.	My fear of failure often spoils the fun at work.
5	Ich bin von meinen Fähigkeiten nicht überzeugt.	I am not convinced of my capabilities.
6 [r]	Ein Misserfolg ist kein Grund für mich, an meinen Fähigkeiten zu zweifeln.	A failure is no reason for me to doubt my abilities.
7	Ich bin meistens unzufrieden mit meinen Arbeitsleistungen.	Mostly, I am dissatisfied with the quality of my work.
8	Sehr gute Resultate Anderer verunsichern mich.	When others achieve very good results, it makes me feel insecure.
9	Ich habe Angst zu scheitern, obwohl ich meistens erfolgreich bin.	I'm afraid to fail, even though I am mostly successful.
10	Wenn ich erfolgreich bin, habe ich häufig das Gefühl, dass ein Misserfolg folgen wird.	When I am successful, I often have the feeling that failure will follow.
11	Mich belasten die hohen Erwartungen Anderer an mich.	The high expectations of others stress me.
	**Arbeitsstil**	**Working Style**
12	Ich erschwere mir vieles, weil ich meine Arbeit aufschiebe.	I make many things more difficult for myself by putting off my work.
13	Oft verschiebe ich das Beginnen wichtiger Aufgaben.	Often I postpone starting important tasks.
14 [r]	Ich fange meine Aufgaben früher an als Andere.	I start my tasks earlier than others do.
15	Ich beende wichtige Aufgaben meistens im letzten Moment.	I usually finish important tasks at the last moment.
16	Ich lenke mich häufig ab, auch wenn ich viel zu erledigen habe.	I often distract myself, even though I have a lot to do.

17 [r]	Ich erledige die wichtigste Aufgabe meistens als erstes.	I usually do the most important tasks first.
	**Entfremdung**	**Alienation**
18	Oft verhalte ich mich unecht.	Often I behave little authentic.
19	Ich fühle mich oft so, als halte ich meine wirkliche Persönlichkeit zurück.	I often feel like I am holding back my true personality.
20	Wirklich ich selbst bin ich nur selten.	I am rarely my real self.
	**Fremd-Selbst Divergenz**	**Other-Self Divergence**
21	Meine Fähigkeiten werden häufig überschätzt.	My skills are often overestimated.
22	Die Erwartungen meiner Kollegen an mich sind zu hoch.	My colleagues' expectations of me are too high.
23	Man überschätzt mich.	People overestimate me.
24	Ich werde häufig für klüger gehalten, als ich bin.	I am often thought to be smarter than I am.
	**Ambition**	**Ambition**
25	Mir ist es sehr wichtig etwas Bedeutendes zu schaffen.	For me, it is very important to create something significant.
26	Eine Arbeit, bei der ich viele Untergebene hätte, würde mich befriedigen.	A job in which I had many subordinates would satisfy me.
27	Bedeutendes zu leisten ist mir im Leben am wichtigsten.	Achieving something significant is what matters most to me in life.
	**Sympathiebedürfnis**	**Need for Sympathy**
28	Mir ist es wichtig sympatisch zu erscheinen.	For me, it is important to appear sympathetic.
39	Mir ist es wichtig gemocht zu werden.	It is important for me to be liked.
30	Ich gelte als sehr hilfsbereite Person.	I am considered a very helpful person.

Note. Original questionnaire in German; translation without psychometric analysis; [r] reversed item; a print version as well as a link to an interactive online version of the questionnaire can be found in the online Ressource 1.

Figure S1

The six-factor CFA model of the IPP30

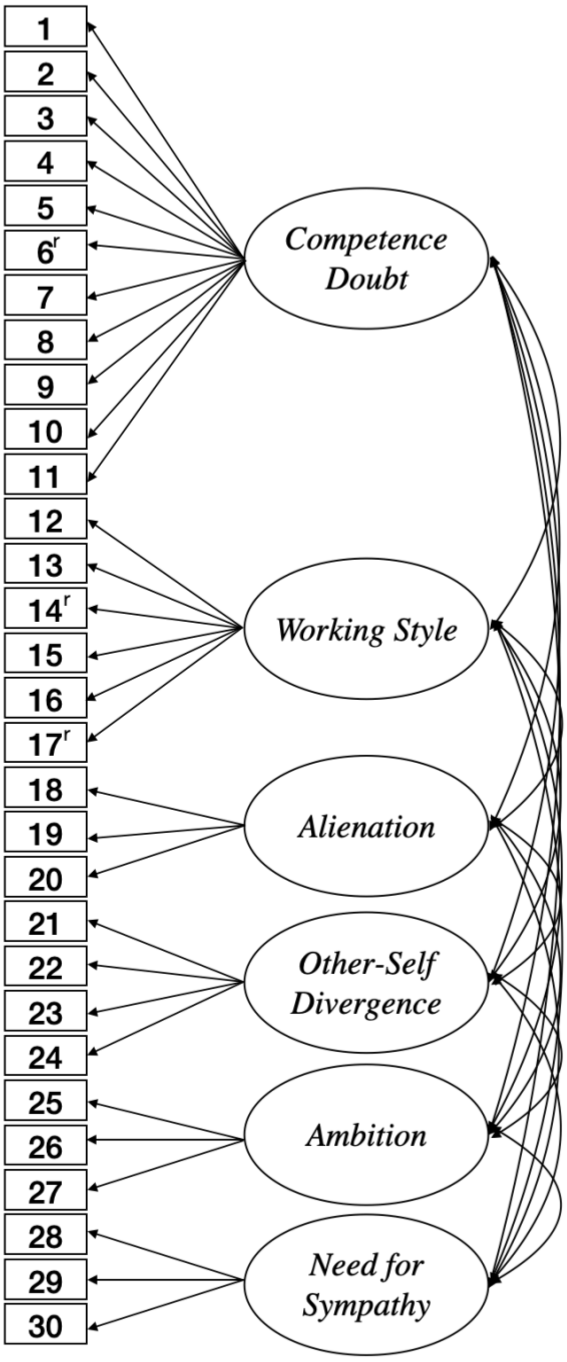

*Note***.** Arrows represent item loadings to the factors; [r] inversed items; Round arrows represent correlations between the subscales.

Figure S2

The one-factor CFA model of the IPP30

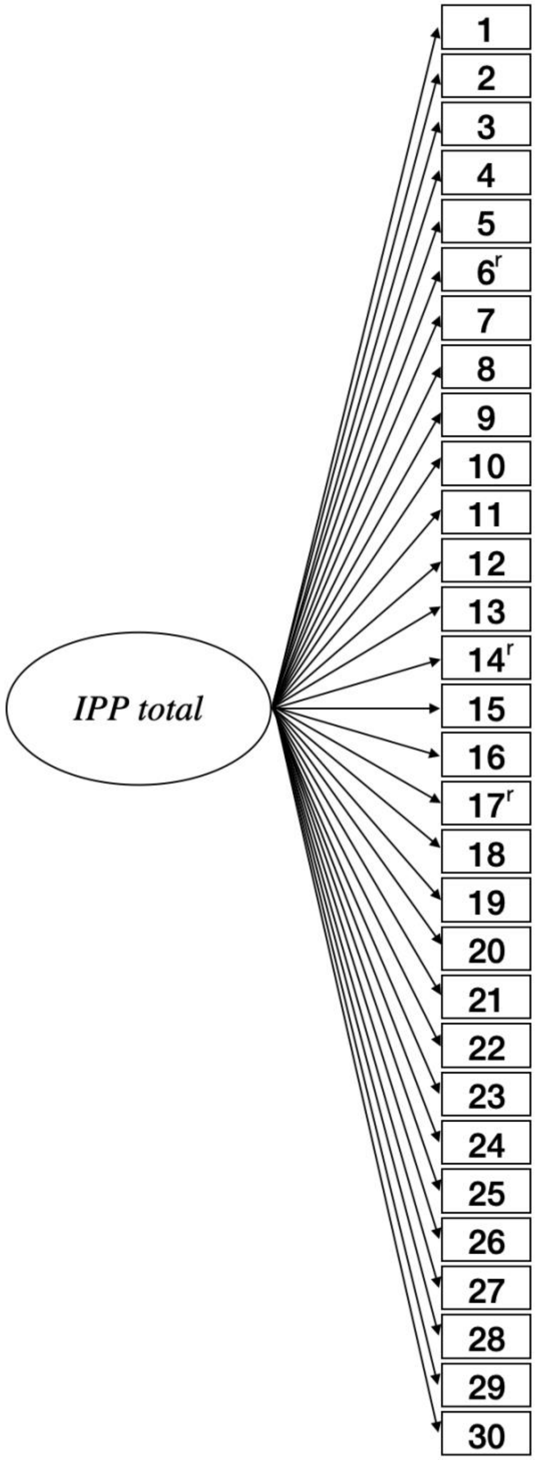

*Note***.** Arrows represent item loadings to the factors; [r] inversed items.

Figure S3

The higher-order factor model of the IPP30

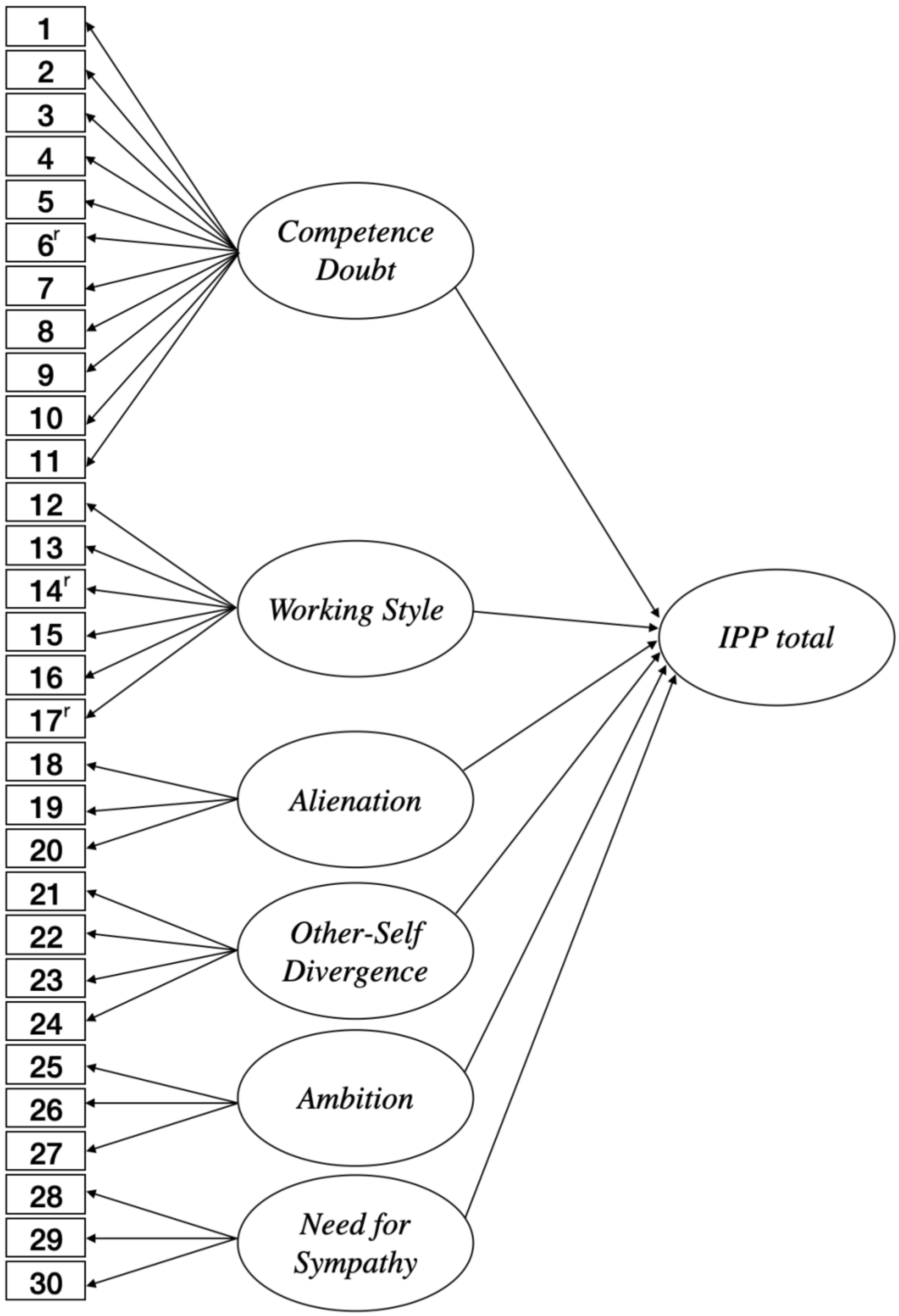

*Note***.** Arrows represent item loadings to the factors; [r] inversed items.
